# Impact of Preoperative Nutritional Status on Postoperative Outcomes of Total Hip and Knee Arthroplasty: A Scoping Review

**DOI:** 10.3390/medicina62050958

**Published:** 2026-05-14

**Authors:** Mariana Garay-Álvarez, Juanita Fetecua-Chaparro, Paula A. Rodríguez-Molina, Giovanni Rodríguez-Rojas, Isabela Álvarez-Rivas, Eduardo Tuta-Quintero, Fernando Ríos-Barbosa, Juan G. Ortiz-Martínez

**Affiliations:** 1 School of Medicine, Universidad de La Sabana, Chía 250001, Colombia; marianagaal@unisabana.edu.co (M.G.-Á.); juanitafech@unisabana.edu.co (J.F.-C.); paularomo@unisabana.edu.co (P.A.R.-M.); giovanniroro@unisabana.edu.co (G.R.-R.); isabelaalri@unisabana.edu.co (I.Á.-R.); 2 Department of Epidemiology, Universidad de La Sabana, Chía 250001, Colombia; eduardotuqu@unisabana.edu.co; 3 Department of Anesthesiology and Perioperative Medicine, Universidad de La Sabana, Chía 250001, Colombia; 4 Department of Orthopedics and Traumatology, Universidad de La Sabana, Chía 250001, Colombia; juan.ortiz7@unisabana.edu.co

**Keywords:** malnutrition, nutritional status, preoperative care, hip arthroplasty replacement, knee arthroplasty replacement, postoperative complications

## Abstract

*Background and Objectives:* Total hip arthroplasty (THA) and total knee arthroplasty (TKA) are widely performed procedures with high success rates but relevant postoperative complications. Preoperative nutritional status is a key modifiable risk factor influencing surgical outcomes. This study aimed to map and synthesize the available evidence on the association between preoperative nutritional status and postoperative complications in patients undergoing primary THA or TKA. *Materials and Methods:* A scoping review was conducted following PRISMA-ScR guidelines. A comprehensive search was performed in PubMed, ScienceDirect, and Scopus, with the last update conducted in April 2026. Studies published between 2015 and 2026 in English and Spanish were included. Eligibility criteria followed the PCC framework. Randomized controlled trials and observational studies were included. Risk of bias was assessed using the Newcastle–Ottawa Scale for observational studies and the Cochrane Risk of Bias tool for randomized trials. *Results:* A total of 1126 records were identified, and 23 studies were included, comprising 447,852 patients. Nutritional status was mainly assessed using serum biomarkers, particularly albumin, followed by anthropometric measures, combined indices, and micronutrients. Poor nutritional status, especially hypoalbuminemia, vitamin D deficiency, and low BMI, was associated with higher rates of infectious complications, prolonged hospital stay, increased readmissions and mortality, and worse functional recovery. *Conclusions:* Preoperative nutritional assessment is essential for perioperative risk stratification in THA and TKA. Integrating biomarkers, indices, and targeted interventions may improve outcomes and reduce postoperative complications.

## 1. Introduction

Total joint arthroplasty (TJA), including total hip arthroplasty (THA) and total knee arthroplasty (TKA), is one of the most common and successful elective surgical procedures worldwide. It is primarily indicated for patients with advanced joint disease, most commonly osteoarthritis (OA), who present with chronic pain, functional limitation, and reduced quality of life despite conservative management [1]. Demand for these procedures is expected to increase substantially as a result of population aging. In the United States, TJA is among the most commonly performed surgical procedures, and its demand is projected to grow in the coming years, with estimates suggesting that the annual volume of TKA procedures may reach up to 3.5 million by 2030 [1,2,3]. Although generally safe, arthroplasty is associated with postoperative complications such as prolonged hospital stay, reoperation, and surgical site infection, thereby generating a significant burden for patients and healthcare systems [2,3,4,5]. Consequently, identifying and modifying preoperative risk factors is essential to optimize outcomes and reduce length of stay (LOS) [6,7].

Preoperative nutritional status has emerged as a crucial and potentially modifiable risk factor associated with adverse outcomes after orthopedic surgery. It refers to the patient’s condition prior to surgery, which can be assessed using serological laboratory values, anthropometric measurements, and standardized nutritional assessments, such as biomarkers, such as serum albumin and other commonly used indicators of nutritional status [1]. Protein–energy malnutrition (PEM), particularly prevalent in older adults, has been linked to increased morbidity, prolonged hospitalization, and higher mortality [5]. Reported malnutrition prevalence in elective arthroplasty ranges from 8.5% to 30%, depending on the screening criteria used [6,8], with higher estimates when Global Leadership Initiative on Malnutrition (GLIM) criteria are applied [9]. Despite growing evidence linking preoperative malnutrition with adverse outcomes after arthroplasty, important limitations remain. Studies have used a wide range of definitions and assessment methods for nutritional status, including serological markers, anthropometric measurements, and other nutritional indicators, leading to substantial heterogeneity across the literature [1]. Furthermore, commonly used biomarkers such as albumin have been widely adopted as proxies of malnutrition despite their known limitations and the lack of consensus regarding optimal cutoff values and predictive accuracy [8]. In addition, evidence suggests that other nutritional factors, such as micronutrient deficiencies, may also influence postoperative outcomes, further contributing to the complexity and fragmentation of the available evidence [2].

Consequently, there is a lack of clarity regarding how preoperative nutritional status is defined, measured, and applied in patients undergoing THA and TKA, as well as its consistent association with postoperative outcomes, highlighting the need to comprehensively map the existing evidence.

Therefore, this scoping review aims to map and synthesize the available evidence on the association between preoperative nutritional status and postoperative complications in patients undergoing THA and TKA, in order to identify knowledge gaps and inform future research and clinical practice.

## 2. Materials and Methods

A scoping review was conducted in September 2025 with the objective of answering the following research question: What is the available evidence on the impact of preoperative nutritional status on postoperative complications in adult patients undergoing major orthopedic surgery (THA or TKA)? The protocol was registered in the Open Science Framework (OSF) on 30 September 2025 (https://osf.io/da28f). The search was systematically updated in April 2026 to ensure the inclusion of the most recent evidence. The update was performed using the same databases (PubMed, ScienceDirect, and Scopus), search strategies (MeSH and DeCS terms combined with free-text keywords and Boolean operators), filters, and eligibility criteria defined in the original search, in order to maintain methodological consistency and reproducibility. All records identified during both the initial search and the update were imported into a systematic review management platform (as detailed below) for screening. Title/abstract screening and full-text assessment were conducted independently by three reviewers, with disagreements resolved through discussion and, when necessary, consultation with a fourth reviewer.

**Figure 1:**
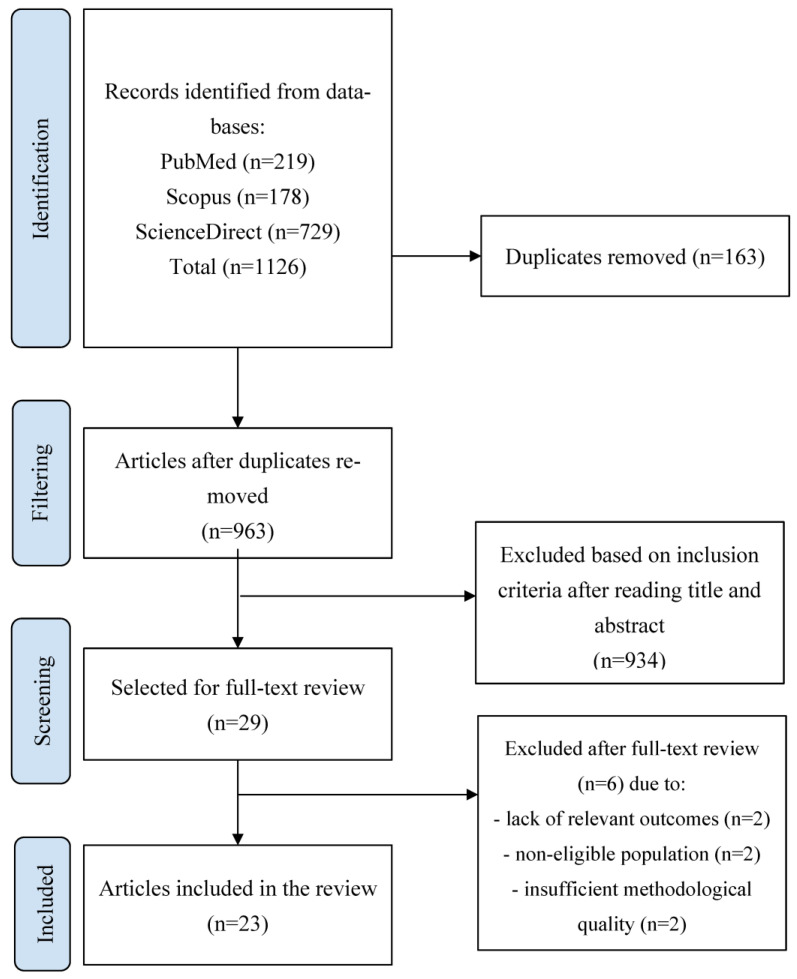
PRISMA Flow Diagram.

In addition, to ensure the reproducibility and rigor of this research, the methodological framework proposed by Arksey and O’Malley was followed, complemented by the Joanna Briggs Institute (JBI) guidelines for scoping reviews [10]. Likewise, the Preferred Reporting Items for Systematic Reviews and Meta-Analyses extension for Scoping Reviews (PRISMA-ScR) was used to guide the reporting of the search process and findings (Figure 1) [11]. Additionally, the PRISMA statement was considered to support the structured synthesis of evidence in exploratory systematic approaches [12].

The methodological process followed the sequential stages proposed by Arksey and O’Malley, consisting of: (a) identification of the research question; (b) identification of relevant studies; (c) study selection; (d) data charting (extraction); and (e) collation, synthesis, and reporting of results. In this context, the guiding research question was defined as: What are the respiratory health consequences of prolonged exposure to disinfectants and antiseptics? The steps for the development of this study are described below.

### 2.1. Identification of the Research Question and Relevant Studies

A comprehensive literature search was conducted using Medical Subject Headings (MeSH) and Health Sciences Descriptors (DeCS) terms related to “*Malnutrition*”, “*Nutritional Status*”, “*Preoperative Care*”, “*Arthroplasty, Replacement, Hip*”, “*Arthroplasty, Replacement, Knee*”, and “*Postoperative Complications*” combined with free-text keywords and Boolean operators (“AND”, “OR”, “NOT”). 

The search was performed in PubMed, ScienceDirect, and Scopus databases, selected for their broad coverage of biomedical literature, allowing a comprehensive and reliable exploration of the available evidence. The complete search strategies and filters applied in each database are detailed in Supplementary Materials Table S1.

Studies published between 2015 and 2026 were included, in English and Spanish, to ensure relevance and contemporaneity of the evidence. The search was updated after protocol registration to incorporate the most recent available studies. The search was last updated in April 2026. The search strategy was repeated in April 2026 using the same databases, search terms, filters, and eligibility criteria applied after protocol registration to identify newly published studies. Title/abstract screening and full-text assessment were performed independently by three reviewers, with disagreements resolved through discussion and consultation with a fourth reviewer when necessary.

### 2.2. Selection Criteria

The article selection criteria were established following the Population, Concept, and Context (PCC) framework recommended by the Joanna Briggs Institute [10].

The population included adult patients (≥18 years) undergoing major orthopedic surgery, specifically total hip arthroplasty (THA) or total knee arthroplasty (TKA). Studies involving pediatric or animal populations, minor orthopedic procedures, spine surgery, fracture- or osteonecrosis-related arthroplasty, revision surgeries, or prosthetic reinterventions were excluded.

The concept focused on studies evaluating preoperative nutritional status and reporting postoperative complications and/or nutritional biomarkers. Studies that did not assess nutritional status or that evaluated nutritional tools in non-surgical populations were excluded.

The context included studies conducted in hospital or surgical settings. Prehospital care, non-clinical contexts, simulation studies, or studies not involving orthopedic patients were excluded.

A wide range of study designs was considered, including clinical trials and observational studies (cohort, case–control, cross-sectional studies, and case series). Editorials, conference abstracts without full-text access, reviews, and studies without original data or clear methodology were excluded. Although systematic reviews were initially identified during the search process, they were not included in the final synthesis, as this scoping review aimed to map and analyze primary studies with original data [10]. The complete selection criteria are detailed in Supplementary Materials Table S2.

### 2.3. Study Selection

After conducting the search and importing the identified records into Rayyan web application, available online (https://www.rayyan.ai/) [13], the study selection process was carried out in three stages:

(1) Initial screening based on titles and abstracts to identify potentially relevant studies, followed by the removal of duplicates.
(2) Independent and blinded full-text assessment by three reviewers according to the predefined inclusion and exclusion criteria. Discrepancies were resolved through discussion and, when necessary, by consultation with a fourth reviewer.
(3) Final selection of studies based on their relevance and alignment with the research objectives.

This rigorous process ensured the consistency and reliability of the included studies. The study selection process is summarized in the PRISMA flow diagram (Figure 1; Supplementary Materials Table S3).

### 2.4. Data Collection, Summary and Presentation of Results

After selecting the fully reviewed articles, data extraction was carried out by creating a table to organize the information. This table includes key data from each selected study, such as bibliographic characteristics (author, year, study design), population characteristics (sample size, age, and sex when available), type of procedure (THA, TKA, or both), methods used to assess preoperative nutritional status (e.g., serum biomarkers, anthropometric measures, or nutritional indices), and reported postoperative outcomes (Table 1).

Data extraction was conducted independently by two reviewers, with discrepancies resolved through discussion. Postoperative outcomes were operationalized as both medical and surgical complications, including infectious complications, length of hospital stay, readmissions, and mortality, as defined in the included studies.

### 2.5. Risk of Bias in Included Studies

**Figure 2:**
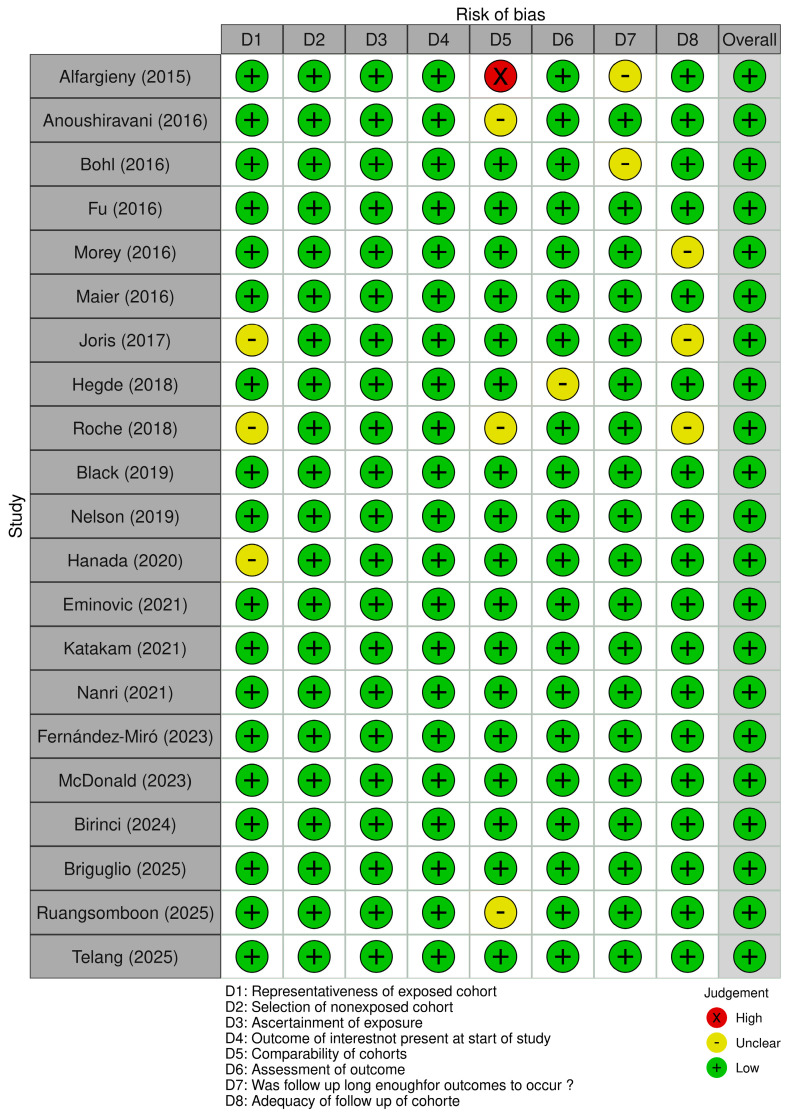
Risk of bias of included cohort studies. The green, yellow, and red dots denote low, unclear, and high risks of bias, respectively [1,2,3,4,5,6,7,8,9,14,15,16,17,18,19,20,21,22,23,24,25].

**Figure 3:**
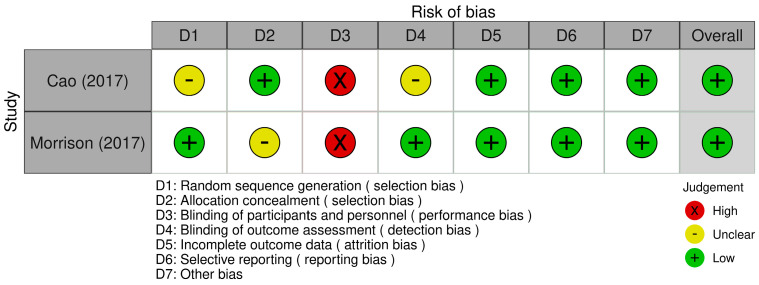
Risk of bias of included randomized controlled trial. The green, yellow, and red dots denote low, unclear, and high risks of bias, respectively [26,27].

The scoping review included observational studies (primarily cohort studies) and randomized controlled trials (RCTs). The risk of bias assessment was conducted according to the study design. For observational studies, the Newcastle–Ottawa Scale (NOS) was applied [12]. This tool evaluates three main domains: selection of study groups, comparability of cohorts, and ascertainment of exposure and outcomes. Specifically, eight items were assessed for cohort studies: representativeness of the exposed cohort, selection of the non-exposed cohort, ascertainment of exposure, absence of the outcome at baseline, comparability of cohorts, outcome assessment, adequacy of follow-up duration, and completeness of follow-up. For randomized controlled trials, the Cochrane Risk of Bias Tool was used [28]. This instrument evaluates seven domains: random sequence generation, allocation concealment, blinding of participants and personnel, blinding of outcome assessment, completeness of outcome data, selective reporting, and other potential sources of bias. The assessment was performed independently by two reviewers, and discrepancies were resolved by a third reviewer. The results of the risk of bias assessment are presented in Figure 2 and Figure 3.

## 3. Results

### 3.1. Study Selection and Characteristics

A total of 1126 records were identified through database searches (PubMed n = 219, Scopus n = 178, ScienceDirect n = 729). After removing 163 duplicates, 963 titles and abstracts were screened, and 23 studies were included (Figure 1). Most studies were retrospective cohorts (n = 19; 82.6%), followed by prospective cohorts (n = 2; 8.7%) and RCTs (n = 2; 8.7%) (Table 1). Regarding surgical procedures, 52% (n = 12) evaluated TKA, 35% (n = 8) THA, and 13% (n = 3) both [1,2,3,4,5,6,7,14,18,19,20,21,22,23,24,25,27,29,30,31,32,33,34].

A total of 447,852 participants were included across the 23 studies, reflecting a large and heterogeneous sample. Study populations varied widely, ranging from small cohorts (n = 48) to large administrative datasets (n = 161,625), reflecting substantial variability in study design and data sources. Age reporting was inconsistent. Approximately half of the studies reported mean age, ranging from 64 to 76.4 years, suggesting that the overall population predominantly consisted of older adults. Across studies with available data, the approximate pooled mean age ranged between 68 and 71 years, consistent with populations typically undergoing arthroplasty or similar interventions. However, a substantial proportion of studies reported age as not recorded (NR), limiting precise aggregate estimation [1,2,3,4,5,6,7,8,9,14,15,16,17,18,19,20,21,22,23,24,25,26,27,29].

Sex distribution was also inconsistently reported. Among studies providing these data, there was a clear predominance of women. Cumulative data suggest approximately 95,652 women versus 60,000 men, corresponding to an estimated distribution of ~61–62% female and ~38–39% male. Several studies showed marked female predominance (e.g., up to 93.5%), whereas a few demonstrated a more balanced or even male-predominant distribution [1,2,3,4,5,6,7,8,9,14,15,16,17,18,19,20,21,22,23,24,25,26,27,29].

The temporal distribution showed a progressive increase in publications over time, with a clear concentration in recent years. The earliest study was published in 2015 (n = 1; 4.3%) [14], followed by a gradual increase: 2016 (n = 5; 21.7%) [1,2,5,15,16], 2017 (n = 3; 13.0%) [23,26,27], 2018 (n = 2; 8.7%) [18,30], and 2019 (n = 2; 8.7%) [8,20]. This trend continued with contributions in 2020 (n = 1; 4.3%) and 2021 (n = 3; 13.0%) [4,6,7,17]. Notably, there is a marked rise in more recent publications, with 2023 (n = 2; 8.7%) and 2025 (n = 3; 13.0%) contributing a substantial proportion of the most current evidence [9,19,22,24,25]. The absence of studies from 2022 may reflect publication lag or selection criteria rather than a true gap in research activity.

### 3.2. Nutritional Assessment Methods and Prevalence of Malnutrition

The prevalence of malnutrition in patients undergoing THA and TKA varied widely depending on the diagnostic criteria used, ranging from 8.5% to 50%. Nutritional status was most commonly assessed using serum protein biomarkers (n = 16; 70%), followed by anthropometric measures (n = 6; 26%), combined indices (n = 4; 17%), and micronutrient assessments (n = 4; 17%) [1,2,3,4,5,6,7,8,9,14,15,16,17,18,19,20,21,22,23,24,25,26,27,29]. Serum albumin was the primary marker in 70% (n = 16) of studies, with hypoalbuminemia most commonly defined as <3.5 g/dL (65%) or <3.0 g/dL (22%). Other frequently used biomarkers included total lymphocyte count (<1500 cells/mm^3^), prealbumin (<16 mg/dL), and transferrin (<200 mg/dL) [5,30]. Combined indices included tools such as the Prognostic Nutritional Index (PNI), Controlling Nutritional Status (CONUT), and GLIM criteria, which demonstrated utility in identifying patients at higher risk of delayed recovery and postoperative complications [7,9,17]. Vitamin D deficiency (<20 ng/mL) was evaluated in 17% (n = 4) of studies [3,5,18,23].

### 3.3. Postoperative Outcomes and Complications

Across studies, hypoalbuminemia was consistently linked to adverse outcomes, including increased infectious complications (31%), prolonged hospital stays (37%), and higher mortality or readmission rates (25%) [1,8,19]. Specifically, patients with albumin levels < 3.5 g/dL face a 1.61 times higher risk of any postoperative complication and a 1.63 times higher risk of major complications, such as cardiac arrest, acute renal failure, and respiratory events [16,19]. Similarly, vitamin D deficiency was correlated with worse postoperative outcomes, including longer hospital stay, higher complication rates, and poorer functional recovery [3,5,18,23,27]. Deficient patients (25D < 20 ng/mL) demonstrated a 1.7 times higher risk of joint stiffness requiring manipulation under anesthesia (MUA), as well as significantly increased rates of deep venous thrombosis (OR 1.80) and myocardial infarction (OR 2.11) [3,19]. Low body mass index (BMI, <18.5–20 kg/m^2^) was also linked to an increased risk of mortality, periprosthetic fracture, sepsis, and greater healthcare utilization, indicating a consistent association between undernutrition and adverse postoperative outcomes [4,9,15,22,24]. Underweight patients undergoing THA exhibit a 2.65-fold increased risk of postoperative anemia and a 3.72-fold higher risk of cardiac complications compared to normal BMI cohorts [15,19].

Infectious complications, particularly surgical site infection (SSI) and periprosthetic joint infection (PJI), were strongly associated with poor nutritional status [1,2,14,19,21]. Preoperative hypoalbuminemia emerged as a robust independent risk factor, with a critical threshold identified at approximately 3.1 g/dL, below which the risk of PJI increased markedly in both THA (OR 1.78) and TKA (OR 1.45) [19]. Additionally, low prealbumin (<16 mg/dL) and transferrin (<200 mg/dL) levels were associated with an approximately twofold increase in infectious and wound complications [21]. Vitamin D deficiency was also linked to higher rates of PJI and a nearly 3-fold higher risk of prosthesis explantation (OR 2.97) within the first postoperative year [3,18].

Beyond infections, malnutrition significantly affected mechanical outcomes and healthcare efficiency [16,19,22]. Patients with low BMI (<20 kg/m^2^) had up to a 5.8-fold higher risk of reoperation and a 7.5-fold higher risk of composite complications, including radiographic abnormalities and persistent pain. These patients also show increased odds for aseptic loosening (OR 1.62) and instability/dislocation (OR 1.84) [22,24]. Length of hospital stay (LOS) was consistently prolonged in malnourished patients; for example, vitamin D deficiency was linked to an average increase of 1.0 to 4.3 hospital days, while patients classified as malnourished by GLIM criteria had longer stays compared to well-nourished individuals [5,7,9,15,17,19,27]. Hypoalbuminemia was associated with staying an average of 1.3 extra days in the hospital and a significantly higher likelihood of discharge to skilled nursing or rehab facilities (30.8% vs. 14.7%) [8,19]. Functional recovery was also impaired, as patients with higher CONUT scores (≥2) experienced a one-day delay in achieving independent ambulation (6 vs. 5 days) and demonstrated lower functional scores even eight years post-surgery [3,7,19].

### 3.4. Mortality and Readmissions

Malnutrition was a strong predictor of short- and mid-term adverse outcomes. Patients with low BMI (<20 kg/m^2^) demonstrated up to an eightfold increased risk of mortality at two years following arthroplasty [4,9,22]. Hypoalbuminemia (<3.5 g/dL) was associated with significantly higher rates of emergency visits and 90-day readmissions, with an optimal predictive cutoff suggested at 3.94 g/dL [6]. Overall, the most frequently reported postoperative outcomes were infectious complications (n = 15; 65%), major medical or surgical complications (n = 12; 52%), length of hospital stay (n = 11; 48%), mortality (n = 6; 26%), and readmissions or surgical revisions (n = 5; 22%) [1,2,3,4,5,6,7,8,9,14,15,16,17,18,19,20,21,22,23,24,25,26,27,29].

### 3.5. Impact of Preoperative Nutritional Optimization

Nutritional interventions demonstrated promising results in reducing postoperative complications [18,19,26]. Preoperative vitamin D supplementation, particularly with high-dose regimens (e.g., 300,000 IU orally), was associated with a reduction in superficial wound infections and postoperative cellulitis [18]. Multimodal nutritional management strategies, including carbohydrate loading and protein supplementation, reduced the need for postoperative albumin transfusions and shortened hospital stay [26]. Importantly, targeted nutritional optimization in malnourished patients reduced the incidence of surgical site infections from 4.4% to 1.3%, underscoring the clinical relevance of preoperative nutritional assessment and intervention [19].

### 3.6. Stratification of Results by Study Design and Effect Size Reporting

Overall, among the 21 cohort studies, the Odds Ratio (OR) was the most frequently reported measure of effect (14/21, 66.7%), followed by Hazard Ratios (HR/aHR) (3/21, 14.3%) and Risk Ratios (RR) (1/21, 4.8%) [1,2,3,4,5,6,7,8,9,14,15,16,17,18,19,20,21,22,23,24,25,26,27,29]. Findings from retrospective cohorts consistently identify hypoalbuminemia and low body mass index as key predictors of adverse outcomes. Serum albumin < 3.5 g/dL is associated with an increased risk of major complications (OR 1.63), surgical site infections (adjusted RR 2.0), and prolonged hospital stay, whereas a BMI < 20 kg/m^2^ is linked to up to an eightfold increase in two-year mortality (OR 8.77) and significantly higher reintervention rates (aHR 5.8) [1,4,8,16,22]. Additionally, observational analyses associate vitamin D deficiency with a 4.3-day longer hospital stay compared with patients with sufficient levels [5,23]. All reported effect measures are detailed in Table 1.

In contrast, randomized controlled trials and interventional studies highlight the beneficial impact of nutritional optimization. However, across the two randomized clinical trials, reporting of effect measures was limited and heterogeneous. For example, implementation of a multimodal nutritional strategy reduced postoperative albumin infusion from 15.2 g to 6.5 g (*p* = 0.006) and shortened hospital stay by 1.7 days (from 5.6 to 3.9 days, *p* < 0.001) [26]. Regarding pharmacological intervention, preoperative administration of a single 300,000 IU dose of vitamin D_3_ in deficient patients reduced the overall complication rate from 8.6% to 4.3% (*p* = 0.005), indicating a protective effect, with an adjusted OR of 15.01 for reducing the risk of superficial wound complications compared with the non-supplemented group [18].

### 3.7. Risk of Bias in the Studies

Overall, the included cohort studies showed a low risk of bias in the selection domains, with appropriately defined and representative populations, adequate selection of exposed and non-exposed groups, and proper exposure ascertainment. However, some studies presented an unclear risk regarding the absence of the outcome at baseline due to insufficient information. Limitations were also identified in cohort comparability and follow-up adequacy, leading to unclear or high-risk ratings, mainly due to insufficient adjustment for confounders, lack of control of relevant factors, and incomplete or poorly reported follow-up data. Regarding the RCTs, most domains were rated as low risk of bias using the Cochrane tool, although a high risk was identified in the blinding of participants and personnel; the remaining domains, including random sequence generation, allocation concealment, outcome assessment blinding, completeness of data, and selective reporting, were adequately addressed.

## 4. Discussion

Preoperative malnutrition is increasingly recognized as a determining factor in arthroplasty outcomes. Even though this kind of association is frequently reported, the majority of studies available are descriptive and subject to residual confounding, which limits causal analysis. The presence of hypoalbuminemia and protein–energy malnutrition is consistently correlated with a higher risk of surgical site infection, periprosthetic joint infection, pneumonia, prolonged hospital stays, and poorer functional recovery [25]. However, there is wide variability in the definitions and diagnostic criteria used, which limit standardization, interpretation, and applicability in nutritional assessment within this study population. 

This heterogeneity is reflected in the use of different cutoff values for key biomarkers (e.g., serum albumin < 3.0 vs. <3.5 g/dL), variability in outcome definitions, and the application of diverse nutritional assessment tools such as PNI, CONUT, and GLIM criteria. Such variability limits comparability across studies, may lead to inconsistent risk classification, and reduces the clinical applicability of findings [6,7,8,9,17,19].

At present, emphasis should be placed on routinely incorporating nutritional assessment and optimization into the perioperative management of patients who are candidates for arthroplasty [16]. Targeted nutritional interventions, such as protein and vitamin supplementation, may promote better functional recovery, reduce postoperative complications, and shorten hospital stay [26].

From a physiological perspective, nutritional status, particularly lean body mass, plays a central role in postoperative recovery. Adequate protein reserves are essential for wound healing and immune competence, as protein deficiencies impair collagen synthesis, fibroblast proliferation, and lymphocyte-mediated immune response, thereby increasing susceptibility to postoperative infections [29,30]. In addition, BMI alone may fail to identify patients with sarcopenic obesity, who may present increased surgical risk despite a normal or elevated BMI.

### 4.1. The Role of Biomarkers in Perioperative Malnutrition

In this context, serum albumin remains the most commonly used biomarker with the greatest accumulated evidence. Low albumin levels (<3.5 g/dL) have been consistently related to increased infectious complications, longer duration of hospitalization, and a higher risk of mortality or readmission [19,20].

Gee et al. [31] conducted a systematic review in which patients with hypoalbuminemia had between 1.7- and 5.9-fold higher risk of surgical site infection and periprosthetic joint infection, as well as twice the risk of severe complications. In addition, hypoalbuminemia was linked to a 30-day mortality of 1.9% and longer hospital stays.

Despite its widespread use, albumin has limitations as an isolated marker, as its concentration can be influenced by various factors, which may reduce its capacity as an indicator of malnutrition. Bala et al. [32], in their cohort study of patients undergoing TJA, demonstrated that albumin is a negative acute-phase protein that also decreases in the presence of inflammation, infection, trauma, or liver disease. Therefore, low albumin levels do not always reflect pure malnutrition, but rather a systemic response to surgical or inflammatory stress or underlying systemic disease; thus, they should be interpreted alongside other nutritional indicators [23,32]. Despite these limitations, serum albumin remains widely used in clinical practice due to its availability, low cost, and consistent association with postoperative outcomes across multiple studies. Its role as a pragmatic marker for risk stratification supports its continued use as part of a broader, multimodal nutritional assessment [1,6,8,26]. In this regard, the combined assessment of serum albumin and C-reactive protein is particularly relevant as it allows differentiation between hypoalbuminemia related to protein–energy malnutrition and that driven by systemic inflammation.

Studies that included micronutrients such as vitamin D (25-OH-D) provide additional evidence on the relationship between specific nutritional deficiencies and postoperative outcomes. Hypovitaminosis D was associated with poorer functional recovery, longer hospital stays, and increased superficial complications, although its relationship with deep infection or the need for reintervention was less consistent [18,23,27]. Brambilla et al. [33] evaluated the relationship between preoperative vitamin D levels and postoperative outcomes in THA and TKA. The wide variability in reported prevalence likely reflects differences in population characteristics and diagnostic thresholds, which may partly account for the inconsistent association with deep infections. For instance, Zha et al. [34] reported a high prevalence of vitamin D deficiency (76.3%) in a prospective cohort of patients undergoing TJA. Such variability underscores the need for standardized definitions and supports the inclusion of micronutrient assessment within a comprehensive perioperative nutritional evaluation [33,34].

### 4.2. Applicability of Nutritional Screening Scales

The variation in the prevalence of malnutrition reported in the literature highlights the lack of consensus regarding the most effective screening method [6], underscoring the interest in the use of combined nutritional indices that integrate biochemical and immunological parameters, such as the Prognostic Nutritional Index (PNI), the CONUT, and the Geriatric Nutritional Risk Index (GNRI) [35]. These indices demonstrated better prognostic performance in identifying patients at increased risk of infection, delayed mobilization, and prolonged hospital stay, even among those with serum albumin levels within the normal range [7,36].

Standardized criteria for the formal diagnosis of malnutrition that integrate phenotypic and etiological criteria, such as the GLIM criteria [9], allow for the detection of moderate or severe malnutrition cases that may not be identified through the use of biomarkers alone. The CONUT score has been applied as a screening tool that integrates serum albumin, total lymphocyte count, and total cholesterol to assess metabolic and nutritional status [5]. The CONUT score has been shown to be a significant risk factor for delayed recovery of mobilization and length of hospital stay after THA [7].

The Charlson Comorbidity Index (CCI) for evaluating chronic medical conditions [4] and the ASA (American Society of Anesthesiologists) score for classifying preoperative physical status and operative risk [6] allow consideration of clinical variables related to malnutrition and adverse postoperative outcomes [4].

From a clinical perspective, the combination of these indices may improve risk stratification and facilitate timely nutritional interventions before surgery, given their simplicity, reproducibility, and low cost [17]. However, the lack of standardized cut-off values and external validation in orthopedic populations limits their applicability. Integrating systematic nutritional screening into preoperative evaluation may strengthen protocols and optimize nutritional status prior to surgery.

### 4.3. Postoperative Complications and Suboptimal Nutritional Status in TJA

Preoperative malnutrition consistently predicts worse postoperative outcomes [2]. The consistent association across a majority of studies suggests a clinically meaningful relationship, although the variability in outcome definitions may influence effect estimates. These findings support a potential dose–response relationship, in which greater degrees of malnutrition may translate into a proportional increase in the risk of adverse events; however, this relationship remains insufficiently explored in prospective designs [24,25]. Patients with protein–energy malnutrition undergoing elective THA have a significantly higher complication rate compared with a control group [6,22].

Among postoperative complications related to malnutrition or hypoalbuminemia, surgical site infection and periprosthetic joint infection are described [2]. Hypoalbuminemia (<3.5 g/dL) has been independently identified as a predictor of a higher risk of surgical site infection in THA [1]. In addition, these infections have been associated with an increased risk of developing pneumonia [1]. Furthermore, malnutrition correlates with longer hospital stay, averaging 0.20 to 1.3 additional days [4]. Malnutrition also affects functional recovery, being a risk factor for delayed early mobilization recovery after THA [7]. Overall, these findings support the hypothesis that poor preoperative nutritional status is related to worse postoperative outcomes in patients undergoing THA and TKA.

Despite the consistency of associations reported, the overall strength of the evidence is limited by the predominance of retrospective designs, heterogeneity in nutritional definitions, and lack of standardized outcome measures. These limitations should be considered when interpreting the observed relationships and highlight the need for more robust and standardized research approaches in this field.

### 4.4. Limitations and Strengths

Our review presents several limitations, as searches were conducted in only three databases, and the restriction to English and Spanish publications may have introduced language bias, potentially excluding relevant evidence from non-indexed or regional literature. In addition, although a risk-of-bias assessment was conducted using the Newcastle–Ottawa Scale and the Cochrane tool, no quantitative synthesis or formal meta-analysis was performed, which limits the ability to draw causal inferences.

Furthermore, the available evidence is characterized by a lack of consensus regarding the definition of malnutrition and the nutritional screening tools used. Commonly employed biomarkers, such as serum albumin or total lymphocyte count, may be influenced by comorbidities and acute inflammatory states, limiting their specificity. In addition, most studies focus on short-term postoperative outcomes, with limited data on long-term functional and orthopedic recovery.

Despite these limitations, this review provides a comprehensive synthesis of the available evidence, integrating diverse nutritional assessment methods and study designs to offer a clinically relevant overview of the impact of preoperative nutritional status on arthroplasty outcomes.

Future research should focus on the standardization of nutritional assessment methods in patients undergoing THA and TKA and on the validation of combined screening tools in orthopedic populations. Prospective studies and RCTs are needed to determine the effectiveness of targeted preoperative nutritional interventions in reducing postoperative complications and improving functional outcomes.

## 5. Conclusions

Preoperative malnutrition is a modifiable and clinically relevant factor influencing postoperative outcomes in patients undergoing THA and TKA. The available evidence shows that poor nutritional status is associated with an increased risk of postoperative complications, including infections, prolonged hospital stays, and impaired functional recovery. The combined use of serum biomarkers, nutritional indices, and standardized screening instruments may provide a more comprehensive preoperative assessment to identify patients at nutritional risk. In particular, the combined use of albumin and C-reactive protein may enhance risk stratification by distinguishing inflammatory from nutritional causes of hypoalbuminemia. Integrating nutritional evaluation into routine preoperative care may support clinical decision-making and contribute to improved surgical outcomes in this population. In this context, malnutrition should be considered a modifiable perioperative risk factor rather than a passive comorbidity.

## Figures and Tables

**Table 1:** General characteristics of the included items.

Author Year (yr)	Study Design	Population (N Age, Sex)	Procedure	Nutritional Assessment	Outcomes	Effect Size	Main Findings
Observational studies							
Bohl et al. (2016) [1]	Retrospective cohort study	N = 101,523; mean age NR; sex NR	THA/TKA	Serum albumin (<3.5 g/dL)	SSI, pneumonia, LOS, readmission	RR 2.0 (SSI), RR 1.5 (complications)	Hypoalbuminemia independently associated with increased risk of postoperative complications
Morey et al. (2016) [2]	Retrospective cohort study	N = 3169; mean age = 69.3 years; Women = 2964 (93.5%); Men = 205 (6.4%)	TKA	Serum albumin and TLC	Wound complications, functional outcomes	OR 1.38 (95% CI: 0.30–6.36)	No association between malnutrition (albumin/TLC) and postoperative wound complications
Hegde et al. (2018) [3]	Retrospective cohort study	N = 6593; mean age NR; Women = 4994 (75.7%); Men = 1599 (24.3%)	TKA	Serum vitamin D (25-OH-D)	Surgical and medical complications	OR 1.69–2.97 (vitamin D deficiency vs. sufficient)	Vitamin D deficiency is associated with higher risk of postoperative complications
Katakam et al. (2021) [4]	Retrospective cohort study	N = 4802; mean age = 65.9 years; Women = 2689 (56%); Men 2113 (44%)	THA/TKA	BMI	Mortality, LOS, readmission, reoperation	OR 8.77 (mortality); +0.44 days LOS	Low BMI independently associated with increased mortality and longer LOS
Maier et al. (2016) [5]	Observational cohort study	N = 1083; mean age = 76.4 years; Women = 567 (52.4%); Men = 516 (47.6%)	THA/TKA	Serum vitamin D (25-OH-D)	LOS	15.6 vs. 11.3 days (*p* = 0.014)	Low vitamin D levels associated with LOS
Eminovic et al. (2021) [6]	Retrospective cohort study	N = 220; mean age = 76.3 years; Women = 144 (65.5%); Men = 76 (34.5%)	THA	Serum albumin and TLC	Complications, LOS, mortality	HR 6.3 (95% CI: 1.7–23.1)	PEM associated with a higher risk of postoperative complications (HR 6.3)
Nanri et al. (2021) [7]	Retrospective cohort study	N = 503; mean age = 64 years; Women = 408 (81.1%); Men = 95 (18.9%)	THA	CONUT score (albumin, lymphocytes, cholesterol)	Time to independent walking, LOS, discharge disposition	HR 0.70–0.74	Preoperative malnutrition associated with delayed mobilization and longer LOS
Black et al. (2019) [8]	Retrospective cohort study	N = 4047; mean age NR; sex NR	THA/TKA	Serum albumin	LOS, 90-day readmission, ED visits, discharge disposition	OR 1.55 (readmission), OR 1.35 (ED visits)	Hypoalbuminemia associated with longer LOS and higher 90-day readmissions and ED visits
Fernández-Miró et al. (2023) [9]	Retrospective cohort study	N= 65; mean age = 75.4 years; Women = 33 (50.8%); Men = 32 (49.2%)	THA	GLIM criteria, serum albumin, TLC, anthropometry	Complications, LOS, discharge	83.3% vs. 33.8% (*p* = 0.028)	Malnutrition associated with increased postoperative complications and longer LOS
Alfargieny et al. (2015) [14]	Retrospective case series	N = 135; mean age NR; sex NR	THA/TKA	Serum albumin and TLC	SSI	3.6 vs. 4.1 g/dL (*p* = 0.011)	Preoperative albumin was the only significant predictor of SSI
Anoushiravani et al. (2016) [15]	Retrospective cohort study	N = 4865; mean age = 70.5 years; Women = 4064 (83.5%); Men = 801 (16.5%)	THA/TKA	BMI	In-hospital complications, LOS, hospital charges, discharge disposition	OR 2.66 (anemia), OR 3.72 (cardiac), OR 4.08–5.91 (complications)	Low BMI associated with higher postoperative complications and increased resource utilization
Fu et al. (2016) [16]	Retrospective cohort study	N = 40,653; age NR; Women = 22,766 (56%); Male = 17,887 (44%)	THA	Serum albumin (<3.5 g/dL) and BMI	Overall complications, major complications, wound complications, LOS, reoperation, mortality	OR 1.61 (any complications); OR 2.35 (respiratory); OR 1.71 (transfusion)	Hypoalbuminemia was a stronger predictor of postoperative complications than obesity
Hanada et al. (2020) [17]	Retrospective cohort study	N = 190; mean age = 74.1 years; Women = 142 (74.7%); Men = 48 (25.3%)	TKA	PNI, BMI, serum albumin, and TLC	Wound complications, PJI	OR 0.858 (PNI); OR 5.10 (infection)	Low PNI associated with postoperative wound complications; PNI predicted complications better than albumin or TLC alone
Birinci et al. (2024) [18]	Multicenter retrospective cohort	N = 1080; mean age = 64.8 years; Women = 861 (79.7%); Men = 219 (20.3%)	THA/TKA	Serum vitamin D (25-OH-D) and supplementation	PJI, wound complications, medical complications, readmission, mortality	8.6% vs. 4.3% (*p* = 0.005); OR 15.01	Vitamin D supplementation reduced overall complications and wound problems, but not PJI
Telang et al. (2025) [19]	Retrospective cohort study	N = 32,952; mean age = 66 years; Women = 19,689 (60%); Men = 13,079 (39%)	THA/TKA	Preoperative serum albumin	PJI, medical complications, surgical complications	OR 1.78 (2.5 vs. 3.1); OR 4.51 (2.0 vs. 3.1)	Preoperative albumin < 3.1 g/dL identified as threshold for increased risk of PJI and complications
Nelson et al. (2019) [20]	Retrospective cohort study	N = 24,586; mean age NR; sex NR	THA	Serum albumin	Mortality and composite perioperative complications	OR 1.86–3.58 (albumin < 3.0 g/dL vs. ≥3.5 g/dL)	Albumin < 3.0 g/dL associated with increased perioperative complications and mortality
Roche et al. (2018) [21]	Retrospective cohort study	N = 161,625; age NR; sex NR	TKA	Serum albumin, prealbumin, transferrin	Infection, wound complications	OR 2.2–2.9 (albumin); OR 1.8–2.3 (others)	Low albumin, prealbumin, and transferrin associated with increased risk of postoperative complications
Ruangsomboon et al. (2025) [22]	Retrospective cohort study	N = 1162; mean age = 68.8 years; Women = 348 (29.9%); Men = 814 (70.1%)	THA/TKA	BMI	Reoperation, composite complications	aHR 5.8 (reoperation), aHR 7.5 (complications)	Low BMI independently associated with increased risk of reoperation and complications
Joris et al. (2017) [23]	Observational cohort study	N = 138; mean age = 72.1 years; Women = 81 (58.7%); Men = 57 (41.3%)	TKA	Serum vitamin D (25-OH-D)	LOS, functional outcome (WOMAC, short- and long-term)	+1.0 day LOS; +5.0 WOMAC (*p* < 0.05)	Vitamin D deficiency is associated with longer LOS and worse long-term functional outcomes
McDonald et al. (2023) [24]	Retrospective cohort study	N = 58,151; mean age = 64.6 years; Women = 35,757 (61.5%); Men = 22,394 (38.5%)	THA	BMI	Revision, sepsis, periprosthetic fracture, dislocation	OR 1.32 (revision), OR 1.51 (sepsis), OR 1.63 (periprosthetic fracture)	Underweight patients had increased risk of revision, sepsis, and periprosthetic fracture
Briguglio et al. (2025) [25]	Prospective cohort study	N = 48; mean age = 71.5 years; Women = 22 (45.8%); Men = 26 (54.2%)	THA	Comprehensive diagnosis of nutritional disorders (undernutrition, sarcopenia, obesity, sarcopenic phenotypes)	Hemoglobin drop, neutrophil response, gait speed recovery	−2.37 g/dL (sarcopenia); −0.80 g/dL (undernutrition); −16.2% neutrophil response	Sarcopenia and undernutrition associated with greater hemoglobin decline and altered inflammatory response; undernutrition influenced functional recovery
Experimental studies							
Cao et al. (2017) [26]	RCT	N = 162; mean age = 66.1 years; Women = 123 (75.9%); Men = 39 (24.1%)	TKA	Multimodal nutritional management (perioperative protein + carbohydrate + early feeding)	Albumin transfusion, electrolyte disorders, LOS, complications	25.9% vs. 43.2% (*p* = 0.021); LOS 3.9 vs. 5.6 days (*p* < 0.001)	MNM reduced albumin transfusion, electrolyte disorders, and hospital stay; improved perioperative albumin and electrolyte levels
Morrison et al. (2017) [27]	RCT	N = 100; mean age NR; sex NR	THA/TKA	Serum vitamin D (25-OH-D) and supplementation	PROMs, LOS, complications	Not reported	Vitamin D deficiency as a potentially modifiable risk factor for poorer postoperative results

BMI, body mass index; CONUT, controlling nutritional status score; LOS, length of stay; PJI, periprosthetic joint infection; PNI, prognostic nutritional index; SSI, surgical site infection; THA, total hip arthroplasty; TKA, total knee arthroplasty; TLC, total lymphocyte count; NR, Not Recorded; OR, Odds Ratio; HR, Hazard Ratio; RR, Relative Risk.

## Data Availability

The data used in this study are available in the included articles.

## Supplementary Materials

The following supporting information can be downloaded at: https://www.mdpi.com/article/10.3390/medicina62050958/s1, Table S1. Search Methods for Each Database. Table S2. Selection Criteria. Table S3. Preferred Reporting Items for Systematic reviews and Meta-Analyses extension for Scoping Reviews (PRISMA-ScR) Checklist [11].

## Abbreviations

The following abbreviations are used in this manuscript:

TJA	Total joint arthroplasty
THA	Total hip arthroplasty
TKA	Total knee arthroplasty
BMI	Body mass index
CONUT	Controlling nutritional status score
LOS	Length of stay
PJI	Periprosthetic joint infection
PNI	Prognostic nutritional index
SSI	Surgical site infection
GLIM	Global Leadership Initiative on Malnutrition
PEM	Protein–energy malnutrition
GNRI	Geriatric Nutritional Risk Index
TLC	total lymphocyte count
OA	Osteoarthritis
OSF	Open Science Framework
JBI	Joanna Briggs Institute
NOS	Newcastle–Ottawa Scale
RCTs	Randomized controlled trials
NR	Not Recorded
